# Not All Runners Cross the Same Finish Line: Sociodemographic Inequalities in COVID-19 Recovery After a Mass Sporting Event

**DOI:** 10.3390/ijerph22091351

**Published:** 2025-08-28

**Authors:** Daniel Martínez-Cevallos, Alejandra Proaño-Grijalva, Frano Giakoni-Ramírez, Daniel Duclos-Bastías

**Affiliations:** 1 Facultad de Cultura Física, Universidad Central del Ecuador, Quito 170129, Ecuador; 2 Universidad Internacional de la Rioja, 26006 Logroño, Spain; 3 Instituto Del Deporte y Bienestar, Facultad de Educación y Ciencias Sociales, Universidad Andres Bello, Las Condes, Santiago 7550000, Chile; 4 iGEO Group, School of Physical Education, Faculty of Philosophy and Education, Pontificia Universidad Católica de Valparaíso, Valparaíso 2362807, Chile

**Keywords:** COVID-19, running, physical activity, sports participation, health promotion

## Abstract

Background: The COVID-19 pandemic significantly disrupted patterns of physical activity and participation in mass sporting events, with recreational runners in Latin America among the most affected. In Colombia, pre-existing inequalities in access to sport further exacerbated these impacts. Nevertheless, evidence on post-COVID-19 impact and recovery experiences among regional runners remains limited. Objective: We examined the sociodemographic profiles, athletic experience, and perceptions of COVID-19-related impact and recovery among participants in the 2023 Medellín Marathon, and to assess differences by educational attainment, employment status, age group, and geographic origin. Methods: A cross-sectional descriptive study was undertaken involving 2486 registered marathon runners. An ad hoc questionnaire assessed COVID-19 symptoms and sequelae, perceived respiratory and physical limitations, fears associated with group exercise, and self-reported recovery. Analyses included descriptive statistics, bivariate comparisons and one-way ANOVA tests. Results: Older participants, retirees and those with lower educational levels reported significantly greater COVID-19 impact, longer recovery periods and higher perceived physical and respiratory limitations. In contrast, younger runners and those with a college education showed more complete physical recovery and attributed protective benefits, such as improved cardiorespiratory function and a lower incidence of respiratory symptoms, to their training. Additionally, runners originating from smaller municipalities and other Latin American countries reported higher levels of impact and lower perceptions of recovery. Conclusions: Post-COVID-19 effects among marathon runners are not uniform but vary according to sociodemographic and contextual factors. These findings underscore the importance of tailored support and readaptation strategies—particularly for vulnerable subgroups—to ensure their safe and equitable return to mass endurance events.

## 1. Introduction

The COVID-19 pandemic, caused by the SARS-CoV-2 virus, marked a global turning point in social, economic, and health-related dynamics, profoundly affecting opportunities and motivations for sport participation across diverse contexts [1,2]. Measures implemented to curb viral spread—such as prolonged lockdowns, suspension of mass events, and restrictions on sports facilities—significantly altered physical activity patterns for millions, leading to increased inactivity, sedentary behavior, and their attendant physical and psychological consequences [3,4].

At the global level, the pandemic’s impact on mental and emotional well-being has been extensively documented [5,6]. The reduction in regular physical activity was associated with significant rises in symptoms of anxiety, depression, stress, and sleep disturbances, affecting both professional athletes and amateurs [6,7,8]. Among recreational runners and sport enthusiasts, this manifested as abandoned or reduced participation and the loss of the social and motivational networks typically built around shared sporting activities [9,10]. The issue was especially acute in populations with specific needs—such as persons with disabilities—who faced not only general restrictions but also additional accessibility barriers and challenges adapting their exercise routines [11,12,13]. Moreover, recent evidence indicates that age and sex differences further modulate these mental health outcomes after COVID-19 recovery among physically active individuals [14].

In Latin America and Colombia, these effects were particularly severe due to existing limitations in sports infrastructure, public policy and unequal access to safe spaces for physical activity [15,16]. Even before the pandemic, significant disparities in sports participation had been identified according to age, gender, and socioeconomic status [17]. Such structural inequalities were exacerbated by confinement measures, disproportionately impacting vulnerable groups and further hindering their ability to maintain an active, healthy lifestyle [18].

Several studies have shown that, despite the gradual reactivation of mass sporting events following the lifting of health restrictions, runners’ return has been uneven and incomplete [19,20]. Fear of infection, post-COVID-19 physical sequelae, and perceived safety concerns in crowded settings have been key factors in this decline in participation, revealing a complex process of readaptation that encompasses both physical and psychosocial dimensions [21,22].

From a theoretical standpoint, the Event Travel Career (ETC) framework posits that sporting-event participation is not an isolated occurrence but part of a progressive trajectory involving the consolidation of athletic identity, accrued experience, and the construction of social meanings [23,24]. Disruptive events such as a pandemic can interrupt or alter this trajectory, leading to drop-out, loss of motivation, or the revision of personal goals [25,26]. Indeed, many recreational runners have since redefined their relationship with running and event participation, prioritizing perceived safety, fear management and new modes of engagement with the activity [12,27].

Although international research has extensively documented COVID-19’s impact on sports participation in Europe, North America, and Asia, there remain substantial gaps regarding its effects on Latin American recreational runners—particularly in structurally unequal contexts like Colombia [16,28]. Mass sporting events such as the Medellín Marathon play a crucial role not only in promoting large-scale physical activity but also in fostering community cohesion, stimulating local economies, and providing spaces for cultural exchange [29,30]. After the pandemic, these events have faced additional health and logistical challenges, requiring organizers to implement biosecurity protocols and to redesign aspects such as capacity controls, distancing measures in start and finish areas, and the management of participants from diverse origins [31,32]. Furthermore, it has been observed that the safe reopening of these races is perceived positively by both runners and spectators, as it contributes to regional economic recovery through sports tourism and strengthens social resilience following [14,33].

Moreover, existing studies tend to focus narrowly on physical impairments, overlooking critical dimensions such as perceptions of recovery, emotional safety when returning to group training, and the interplay between socioeconomic status and reintegration into mass sporting events [34,35].

In this context, mass sporting events such as the Medellín Marathon represent ideal settings in which to comprehensively examine post-COVID-19 impact and recovery dynamics among runners. Beyond their competitive value, these events contribute significantly to social cohesion, the generation of well-being, and the promotion of active lifestyles [27,36]. Consistent with United Nations Educational, Scientific and Cultural Organization perspective [37], sport should be understood not merely as a recreational or leisure activity but as a strategic tool for the emotional, social, and economic recovery of affected populations.

It is important to note that, although recreational running is generally low-cost, destination marathons like the Medellín Marathon involve high entry fees, travel expenses, and the cost of specialized equipment. This can limit participation among those with higher disposable incomes, introducing a socioeconomic bias into the sample.

Therefore, this study aims to analyze the sociodemographic profiles, athletic experiences and perceptions of post-COVID-19 impact and recovery among participants in the 2023 Medellín Marathon, assessing significant differences by educational attainment, age, employment status, and geographic origin. By doing so, we seek to furnish scientific evidence to inform the design of targeted prevention, readaptation, and support interventions in mass-participation sporting contexts across Latin America, thereby strengthening the resilience and well-being of the running community in the post-pandemic era.

## 2. Methods

### 2.1. Study Design

A descriptive, cross-sectional, comparative study with a quantitative approach was conducted to examine the sociodemographic characteristics and the post-COVID-19 impact and adaptation among runners participating in the 2023 Medellín Marathon. Differences were evaluated according to educational level, employment status, age group, and geographic origin.

### 2.2. Participants

The study sample comprised 2488 runners—out of approximately 18,000 total entrants in the 2023 Medellín Marathon—who voluntarily agreed to participate. A non-probability convenience sampling method was used, including only those who completed the online questionnaire provided by event organizers. Of these respondents, 56.6% were male and 43.4% were female. Age distribution covered three categories: up to 30 years (19.3%), 31–45 years (56.6%), and 46 years or older (24.2%). The vast majority resided in Colombia (96.6%), with 41.5% reporting Medellín as their place of origin.

### 2.3. Instrument

A custom-designed, self-administered questionnaire was developed and deployed to capture the following dimensions:

It is important to mention that in Table 1, in the ‘Athletic Experience’ dimension, it has been categorized according to criteria used in previous Latin American studies [38,39,40]. Also, in the sociodemographic profile dimension, the “Knowledge of Social Causes” section asked whether the runner participated in the Medellín Marathon as part of a social or charitable cause (e.g., fundraising for NGOs, charity teams, awareness campaigns). The response was dichotomous (yes/no).

### 2.4. Procedure

Data were collected during the week following the 2023 Medellín Marathon via an online questionnaire disseminated to all registered participants through the event’s official organizers. Prior to data collection, runners received an informational email outlining the study’s objectives, academic purpose, and conditions for participation; those who agreed to participate provided electronic informed consent. Anonymity was strictly maintained: all responses were recorded directly in the LimeSurvey platform without linkage available to email addresses or any personally identifiable information. The event organizers alone managed the database, ensuring that the data were used exclusively for academic research purposes.

The ad hoc questionnaire was designed and administered in Spanish, given that 96.6% of the participants were native Spanish speakers. For the small group of English-speaking foreign athletes (approximately 3% of the total), field staff offered assistance with oral translation into English only upon request by the participant. All responses were recorded in Spanish, so no back-translation was necessary.

### 2.5. Data Analysis

Statistical analyses were performed using IBM SPSS Statistics version 25. Descriptive statistics (frequencies, percentages, means and standard deviations) characterized the sample and summarized participants’ perceptions. One-way analyses of variance (ANOVAs) were then conducted to assess significant differences in impact and recovery perceptions across educational level, employment status, age group, and geographic origin. When ANOVA results were significant, pairwise comparisons were carried out using Tukey’s HSD or Tamhane’s T2 post hoc tests, as appropriate, with a significance threshold set at *p* < 0.05. Tukey’s HSD was applied when Levene’s test indicated homogeneity of variances (*p* > 0.05), whereas Tamhane’s T2 was used in cases of unequal variances (Levene’s test *p* < 0.05).

## 3. Results

### 3.1. Sociodemographic Profile

The information on the socio-demographic profile of the sample can be found in Table 2. The gender distribution revealed a slight male predominance (56.6% male vs. 43.4% female). Regarding age, 56.6% of participants were aged 31–45 years, 24.2% were 46 years or older and 19.3% were 30 years or younger. With respect to running experience, 44.8% were classified as beginners (1–5 events), 38.9% as intermediate (6–20 events), 11.8% as confirmed runners (21–50 events), and 4.4% as experts (>50 events).

The majority (55.3%) reported that the 2023 Medellín Marathon was their first instance of participation, whereas 44.7% had competed in previous editions. Notably, 80.1% of respondents indicated they were unaware of the social causes promoted by the marathon organization. Of the 2488 runners, 496 (19.9%) reported running in support of a social cause. When comparing by municipality of origin, 22.5% of runners from small municipalities reported participating in causes, compared to 15.2% of participants from Medellín.

In terms of employment status, 79.1% were employed full-time, 11.0% were employed part-time, 3.7% were unemployed, 2.8% were students and 3.4% were retired. Regarding educational attainment, 92.2% held a university degree, 7.5% completed secondary education, and 0.2% had primary education. With respect to geographic origin, 96.6% were Colombian nationals, 2.8% originated from other South or Central American countries, and 0.6% came from other international regions. Finally, 41.5% of participants resided in Medellín, 56.1% resided in other Colombian municipalities, and 2.5% resided abroad.

### 3.2. Post-COVID-19 Impact and Adaptation Profile

A sizeable proportion of participants reported a prior SARS-CoV-2 infection; however, most indicated they had fully recovered without significant sequelae. Analysis of time since infection showed that fewer than 10% contracted COVID-19 within the six months preceding the marathon (Table 3), whereas a majority had experienced the illness more than one year earlier, suggesting that most runners faced the event without recent post-infection effects.

Regarding post-infection rehabilitation, most of runners reported no need for medical or therapeutic intervention, indicating a clinically stable cohort with no major complications. This stability was mirrored in self-assessments of training capacity: most participants denied that COVID-19 had impeded their preparation, minimizing any perceived functional impact of the virus on their athletic training.

On the psychosocial front, perceived safety when running in groups was high—few participants expressed fear of resuming collective activities, an important consideration given ongoing concerns about crowd-related transmission. Likewise, most runners reported no decline in physical performance attributable to COVID-19, reflecting positive self-appraisals of their fitness levels.

Respiratory health—often cited as a potential post-COVID-19 concern—also appeared largely unaffected: the majority did not experience breathing limitations, and overall physical recovery was deemed satisfactory by most, indicating a return to pre-infection capacities.

Notably, approximately one quarter of participants credited their training regime with aiding recovery from COVID-19 sequelae, and nearly one third perceived that regular exercise may have conferred a protective effect against reinfection.

Overall, more than half of the runners felt fully recovered at the time of competition, and most reported no persistent COVID-19-related issues. Nevertheless, a minority did report ongoing physical or emotional limitations, underscoring the need for targeted support and intervention strategies for these at-risk individuals.

### 3.3. Comparisons According to Post Hoc Tests

The post hoc analyses for one-way ANOVA were as follows: Tukey’s HSD was used when the assumption of equal variances was met (Levene’s test *p* > 0.05). In cases of heterogeneity of variances (Levene’s test *p* < 0.05), the Games–Howell test was employed for pairwise comparisons.

#### 3.3.1. Post Hoc Comparisons by Educational Level

One-way ANOVA followed by post hoc analyses identified significant differences according to educational attainment. Specifically, participants with a university degree reported a lower prevalence of prior COVID-19 infection and a shorter time since their last infection compared to those with only secondary education (both comparisons *p* < 0.001). This pattern suggests that higher educational level may confer protective effects through more effective prevention and self-care practices. The results of this test can be found in detail in Table 4.

#### 3.3.2. Comparisons by Employment Status

Table 5 shows significant differences were identified when comparing groups by employment status. Retired runners exhibited a higher incidence of prior COVID-19 infection, a longer time since illness onset, and greater difficulties related to fear of group running and perceived physical recovery compared with full-time workers, part-time workers, and students (*p* < 0.05). These findings indicate that both age and employment status influence the post-COVID-19 impact and recovery experience among runners.

#### 3.3.3. Comparisons by Age Group

Multiple comparison analyses indicated significant differences among age groups across various post-COVID-19 impact and perception variables. First, as shown in the Table 6, runners aged ≥46 years showed a higher frequency of having contracted COVID-19 compared with both the ≤30-year-old group and the 31–45-year-old group (*p* < 0.001 for both). Regarding time since illness, participants aged ≥46 years reported having experienced COVID-19 longer ago than the younger cohorts, with significant differences versus the ≤30 and 31–45-year-old groups (*p* < 0.001 for both).

In terms of perceived fear when running in groups, older adults reported higher levels of fear than both younger and middle-aged runners, with differences reaching significance against the ≤30-year-old group (*p* < 0.001) and the 31–45-year-old group (*p* < 0.05). Conversely, respiratory impact was greater among the ≤30 and 31–45-year-old cohorts than among those aged ≥46 years (*p* < 0.001 for both comparisons).

Concerning perceptions of training’s protective effects, runners aged ≥46 years were more likely to agree that physical training helped shield them from COVID-19 infection than both younger and middle-aged participants (*p* <0.001). Finally, the ≤30 and 31–45-year-old groups reported significantly higher perceptions of being fully recovered after COVID-19 compared with the ≥46-year-old group (*p* < 0.001 for both).

These findings suggest that age influences both the experience of having had the disease and perceptions related to training, recovery, and potential respiratory sequelae.

#### 3.3.4. Comparisons by Geographic Origin

Post hoc analyses, shown in Table 7, revealed significant differences between origin groups in several variables related to post-COVID-19 impact and perception. First, runners originating from Colombia reported having contracted COVID-19 in a lower proportion than those from other South and Central American countries, a difference that was statistically significant (*p* < 0.05).

Likewise, the group of Colombian runners indicated having faced fewer impediments to carrying out their training compared with participants from other regional countries (*p* < 0.05), which may suggest better adaptation or greater availability of resources for sports practice during and after the pandemic.

Regarding the need for rehabilitation following infection, it was found that runners from countries outside Colombia and South America exhibited lower levels of rehabilitation requirement compared with both Colombian runners (*p* < 0.001) and those from South/Central America (*p* < 0.05). This finding suggests a possible difference in the severity of impact or in recovery strategies according to origin.

Finally, Colombian runners were less likely to consider that training had helped them overcome the sequelae of the illness compared with runners from other South American countries (*p* < 0.05). This result may relate to differences in the perception of training’s protective role or in how each group experienced the consequences of COVID-19.

#### 3.3.5. Comparisons by Municipality

Post hoc analyses identified significant differences among groups based on municipality of origin. First, Medellín runners reported less fear when running in large crowds compared with those from other Colombian municipalities (*p* < 0.01). Likewise, this group also exhibited a lower perception that training helped overcome COVID-19-related sequelae, both in comparison with runners from other municipalities (*p* < 0.001) and with those from abroad (*p* < 0.01).

Similarly, participants from Medellín were less likely to perceive that training had a protective effect against COVID-19 infection, when compared with runners from other Colombian municipalities (*p* < 0.001) and from other countries (*p* < 0.05). These results suggest a lower perceived vulnerability and reduced need for adaptation among Medellín runners relative to the other groups. Both means and p-values can be found in Table 8.

## 4. Discussion

The findings of the present study show that, despite the high proportion of runners who contracted COVID-19 (52.7%), most of participants in the 2023 Medellín Marathon reported a positive perception of their physical, emotional, and functional recovery. These results reinforce the protective role of regular physical exercise in the post-pandemic context, as previously highlighted by research demonstrating its capacity to mitigate the psychological, physiological, and social impacts of confinement [4,9]. In the same vein, studies such as that by Lesser and Nienhuis [21] showed that individuals who maintained regular levels of physical activity during the pandemic experienced higher levels of well-being and fewer symptoms of anxiety and depression, underscoring the importance of exercise as a coping strategy in the face of the global disruption caused by COVID-19.

However, significant differences were identified according to sociodemographic variables, indicating that the post-COVID-19 experience was not homogeneous among runners. For example, participants aged 46 years and older reported a higher frequency of having contracted the disease and greater fear of participating in mass events, which aligns with studies indicating increased social anxiety and risk perception in older adults resuming large-scale sporting activities [10,17]. These age-related differences have also been documented in systematic reviews—such as Wunsch et al. [41]—which highlight a significant reduction in physical activity levels among older adults during the pandemic, compounded by increased physical and psychosocial barriers to resuming exercise in the post-COVID context.

Similarly, retirees and unemployed runners reported greater respiratory and physical difficulties, a pattern that may be attributed to lower prior exposure to regular training and greater social and economic vulnerability, as suggested by evidence on structural inequalities in sports access [16,19]. Delbressine et al. [42] provide additional support for this, showing that post-COVID-19 syndrome had a more pronounced impact on populations with lower baseline functional capacity, who also reported reductions in perceived physical activity and prolonged physical sequelae.

On the other hand, educational level emerged as a relevant differentiator. Runners with a university education not only reported a lower prevalence of infection but also a faster recovery time, which may be related to greater access to health resources, preventive information, and continuity in physical activity routines during lockdown [15,18]. Consistent with this, higher-educated populations have been shown to exhibit greater adherence to healthy behaviors in health crisis contexts [9]. Furthermore, Hagiwara et al. [43] found that student-athletes who maintained a strong athletic identity during the pandemic demonstrated greater emotional resilience, suggesting a positive interaction between formal education and athletic identity in post-COVID recovery.

From a psychosocial perspective, more than half of the runners considered that their marathon training contributed to their recovery or even acted as a protective factor. This finding relates to the Event Travel Career model [23], which views sports event participation as part of a motivational trajectory. In this sense, the marathon functioned not only as a sporting goal but also as a catalyst for physical and emotional resilience, facilitating positive coping with the lingering effects of COVID-19 [24,26]. In line with this idea, Mosqueira-Ourens et al. [44] observed that recreational runners returning to activity after prolonged confinement experienced feelings of well-being, accomplishment, and reclamation of their athletic identity, despite initial physical challenges.

However, the analysis by geographic origin also revealed significant gaps. Runners from outside Medellín or from other countries reported higher levels of perceived impact, which may be linked to contextual differences in pandemic management, access to training facilities, or institutional support for physical activity. Studies of sporting events in urban settings have underscored the importance of civic capital and community networks as key elements for facilitating a safe return to exercise [9,36]. These territorial disparities were likewise identified by Lesser and Nienhuis [21], who demonstrated that local policies governing access to public spaces during the pandemic directly influenced the resumption of physical activity.

In line with these findings, recent research has highlighted the need to promote inclusive sports policies that address post-pandemic conditions and account for the diverse experiences and perceptions of participants [20,34]. For instance, Tokac et al. [9] found that, for runners with visual impairments, the pandemic exacerbated barriers to safe participation, underscoring the necessity of accessible environments and inclusive innovations at mass events. This evidence stresses the importance of incorporating accessibility and equity criteria into event planning—particularly in Latin American contexts, where social inequalities are more pronounced.

Finally, it is important to recognize that events such as the Medellín Marathon serve not only recreational or competitive purposes but also social, cultural, and public health functions. Their role in rebuilding the social fabric has been emphasized by organizations such as UNESCO [45], which advocates using sport as a tool for emotional and community recovery. In this regard, the present study offers empirical evidence on post-COVID-19 vulnerability and adaptation profiles, providing a valuable basis for designing targeted interventions to ensure equity, accessibility, and sustainability in urban recreational sport across Latin America.

Building on our results, several practical implications emerge that can inform future policies and practices. First, public health authorities and sporting event organizers should prioritize targeted outreach and support for subgroups identified as most vulnerable—namely, older runners, individuals with lower educational attainment, retirees, and participants from smaller municipalities or other Latin American countries. For instance, pre-race screening protocols and post-race follow-up programs could include focused education on self-care strategies, tailored return-to-training guidelines, and easily accessible telehealth resources for those who report ongoing respiratory or physical limitations. Second, training clubs and coaches can use these findings to develop differentiated training regimens: younger and university-educated runners may benefit from group sessions emphasizing protective factors, whereas more vulnerable participants might require modified intensity, extended recovery periods, and closer medical supervision. Third, local governments and regional sports federations should consider allocating funding to subsidize rehabilitation clinics or community-based exercise programs in underserved areas, thereby mitigating geographic disparities in access to post-COVID-19 support. Moreover, marathon organizers could integrate digital monitoring tools—such as mobile apps for tracking symptoms and recovery progress—that automatically flag runners at higher risk, prompting personalized follow-up. Finally, these insights could shape national policies by reinforcing the importance of vaccination campaigns tailored to recreational athletes and by advocating for standardized guidelines on safe reintegration into endurance events. Collectively, such measures would not only enhance runner safety and well-being but also strengthen the resilience of mass sporting events against future public health challenges.

Additionally, several limitations should be acknowledged. First, the cross-sectional design precludes any inference of causality between sociodemographic factors and COVID-19 recovery outcomes; longitudinal studies would be necessary to examine temporal changes in physical and respiratory recovery trajectories. Second, all data were self-reported, which introduces the possibility of recall bias—participants may have inaccurately recalled the timing or severity of their COVID-19 symptoms and recovery experiences. Third, the reliance on an ad hoc questionnaire without concurrent validation against objective physiological measures (e.g., pulmonary function tests or wearable device data) limits the ability to verify self-reported limitations. Fourth, the study sample was drawn exclusively from registrants of the 2023 Medellín Marathon, so findings may not generalize to runners who did not participate or to broader populations of endurance athletes in other regions. Fifth, potential confounders such as vaccination status, comorbidities, and differences in access to medical care were not controlled for, which could influence both COVID-19 impact and recovery perceptions. Finally, while we captured geographic origin, we did not assess neighborhood-level socioeconomic indicators or environmental factors (e.g., air quality, availability of green spaces) that might further explain regional disparities.

Future research should consider several directions. A prospective cohort design could track runners from pre-infection through recovery, incorporating objective physiological assessments and standardized functional tests to validate self-reported data. Intervention studies—such as randomized controlled trials of graded return-to-run protocols—would help identify best practices for safe reintegration into endurance training. Qualitative investigations (e.g., focus groups or in-depth interviews) could explore psychosocial dimensions of recovery, including motivation, social support, and perceived barriers among different subgroups. Expanding the geographic scope to include multiple cities or countries across Latin America would allow comparison of how local healthcare infrastructure and pandemic policies influenced recovery outcomes. Additionally, examining the role of vaccination timing, booster status, and post-acute sequelae of SARS-CoV-2 infection (PASC) would provide a more nuanced understanding of long-term recovery patterns. Finally, future studies should assess the impact of virtual or hybrid mass-event formats on both physical recovery and mental well-being, given the continuing evolution of sporting events in a post-pandemic context.

## 5. Conclusions

The results of this study demonstrate that post-COVID-19 impact and recovery among recreational runners have not been uniform but exhibit significant variations according to sociodemographic and contextual factors. Advanced age, retirement status, lower educational attainment, and origin from municipalities other than Medellín or from other Latin American countries are associated with greater perceived physical and respiratory impact and with lower perceptions of recovery, even after the resumption of mass sporting events.

These findings underscore the importance of acknowledging the heterogeneity within runner populations and the need to design support and physical readaptation strategies that account for these differences. There is a clear necessity to focus interventions on those runners who, due to their socioeconomic status or employment situation, have faced greater barriers during and after the pandemic. Moreover, the positive perceptions reported by those who maintained higher training levels or who have an established athletic background suggest that regular physical activity not only contributes to physical recovery but also strengthens resilience in the face of crises.

In this regard, mass sporting events in Latin America—such as the Medellín Marathon—not only present an opportunity to promote active lifestyles but also to identify and address inequities in post-pandemic recovery. This study provides empirical evidence that can serve as a foundation for event organizers, sports institutions, and public policymakers to develop prevention, readaptation, and health-promotion programs tailored to the sociocultural and territorial realities of the region’s runners. Furthermore, nearly one in five runners ran for a social cause, highlighting the importance of integrating community initiatives into future events to enhance post-COVID-19 psychosocial recovery.

Future research should delve deeper into the relationship between specific sequelae (physical and psychosocial) and training adherence, as well as the effectiveness of intervention strategies implemented in post-pandemic sporting contexts, thereby contributing to the enhancement of runners’ physical, emotional, and community health.

## Figures and Tables

**Table 1:** Structure of the applied questionnaire.

Section	Dimension	Description	Variable Type
1	Sociodemographic Profile	Collects basic participant information: gender, age group, educational level, employment status, social causes awareness, and country and municipality of origin.	Categorical; Ordinal
2	Athletic Experience	Race history (number of previous events) and first participation in the Medellín Marathon are evaluated. Races are also categorized as follows: beginner—1 to 5 events completed at any distance; intermediate—6 to 20 events completed; and advanced—more than 20 events completed.	Categorical
3	Post-COVID-19 Impact	Evaluates COVID-19 incidence, time since infection, symptoms, respiratory limitations, need for rehabilitation and perceived recovery.	Ordinal; Likert-type
4	Training Impact	Measures perceptions of the effect of training on recovery and prevention related to COVID-19.	Likert-type
5	Psychosocial Aspects	Includes items on fear of group running, perceived performance and post-event psychosocial challenges in the post-pandemic context.	Likert-type

Note: Section 3, Section 4 and Section 5 used a 5-point Likert scale (1 = strongly disagree; 2 = disagree; 3 = neither agree nor disagree; 4 = agree; 5 = strongly agree).

**Table 2:** Sociodemographic profile of participants.

Variable	Category	*n*	%
Gender	Male	1406	56.6
	Female	1080	43.4
Age	≤30 years	479	19.3
	31–45 years	1407	56.6
	≥46 years	601	24.2
Running Experience	Beginners (1–5 events)	1113	44.8
	Intermediate (6–20 events)	966	38.9
	Confirmed (21–50 events)	294	11.8
	Expert (>50 events)	110	4.4
First-time Participation	Yes	1375	55.3
	No	1113	44.7
Social Causes Awareness	Yes	496	19.9
	No	1992	80.1
Employment Status	Full-time	1967	79.1
	Part-time	274	11.0
	Unemployed	93	3.7
	Student	69	2.8
	Retired	85	3.4
Education Level	Primary	6	0.2
	Secondary	187	7.5
	University	2295	92.2
Country of Origin	Colombia	2404	96.6
	Other South/Central America	69	2.8
	Other country	15	0.6
Municipality of Origin	Medellín	1032	41.5
	Other municipality	1395	56.1
	Outside Colombia	61	2.5

Note: Percentages are calculated from valid cases (*n* = 2488).

**Table 3:** Post-COVID-19 impact and adaptation among marathon participants.

Variable	Category	*n*	%
Time since infection	<6 months	193	7.8
	6 months–1 year	453	18.2
	>1 year	713	28.7
	Never infected	1129	45.4
Needed rehabilitation	Never	2195	88.2
	Almost never	104	4.2
	Sometimes	72	2.9
	Almost always	49	2.0
	Always	68	2.7
Perceived training impediment	Strongly disagree	1793	72.1
	Disagree	229	9.2
	Neither agree nor disagree	190	7.6
	Agree	119	4.8
	Strongly agree	157	6.3
Fear of group running	Never	1740	69.9
	Almost never	292	11.7
	Sometimes	241	9.7
	Almost always	107	4.3
	Always	108	4.3
Perceived performance decline	Strongly disagree	1706	68.6
	Disagree	254	10.2
	Neither agree nor disagree	220	8.8
	Agree	159	6.4
	Strongly agree	149	6.0
Respiratory limitations	Never	1877	75.4
	Almost never	213	8.6
	Sometimes	145	5.8
	Almost always	111	4.5
	Always	142	5.7
Difficult recovery	Strongly disagree	1889	75.9
	Disagree	212	8.5
	Neither agree nor disagree	178	7.2
	Agree	102	4.1
	Strongly agree	107	4.3
Training aided recovery	Strongly disagree	1174	47.2
	Disagree	125	5.0
	Neither agree nor disagree	271	10.9
	Agree	292	11.7
	Strongly agree	626	25.2
Training perceived as protective	Strongly disagree	883	35.5
	Disagree	163	6.6
	Neither agree nor disagree	329	13.2
	Agree	347	13.9
	Strongly agree	766	30,8
Feel fully recovered	Strongly disagree	885	35.6
	Disagree	38	1.5
	Neither agree nor disagree	104	4.2
	Agree	168	6.8
	Strongly agree	1293	52.0
Experienced issues but still competed	Strongly disagree	2126	85.5
	Disagree	95	3.8
	Neither agree nor disagree	78	3.1
	Agree	67	2.7
	Strongly agree	122	4.9

Note: Percentages are calculated from valid cases (*n* = 2488).

**Table 4:** Differences by educational level.

Variable	Comparison	MD	*p*
History of COVID-19 infection	Secondary vs. university	0.17	***
Time since infection	Secondary vs. university	0.40	***

Note: *** *p* < 0.001. MD = mean difference; *p* = significance.

**Table 5:** Differences in Post-COVID-19 variables by employment status.

Variable	Comparison	MD	*p*
Variable	Comparison	MD	***
History of COVID-19 infection	Retired vs. full-time	0.25	**
History of COVID-19 infection	Retired vs. fart-time	0.22	**
Time since infection	Retired vs. full-time	0.40	*
Fear of group running	Students vs. full-time	0.27	*

Note: * *p* < 0.05; ** *p* < 0.01; *** *p* < 0.001. MD = mean difference; *p* = significance.

**Table 6:** Differences by age group.

Variable	Comparison	MD	*p*
History of COVID-19 infection	≥46 vs. ≤30 years	0.15	***
History of COVID-19 infection	≥46 vs. 31–45 years	0.14	***
Time since infection	≥46 vs. ≤30 years	0.20	***
Time since infection	≥46 vs. 31–45 years	0.21	***
Fear of group running	≥46 vs. ≤30 years	0.30	***
Fear of group running	≥46 vs. 31–45 years	0.14	*
Respiratory limitations	≤30 vs. ≥46 years	0.21	***
Respiratory limitations	31–45 vs. ≥46 years	0.19	***
Training perceived as protective	≥46 vs. ≤30 years	0.48	***
Training perceived as protective	≥46 vs. 31–45 years	0.33	***
Feel fully recovered	≤30 vs. ≥46 years	0.34	***
Feel fully recovered	31–45 vs. ≥46 years	0.36	***

Note: * *p* < 0.05; *** *p* < 0.001. MD = mean difference; *p* = significance.

**Table 7:** Differences by geographic origin.

Variable	Comparison	MD	*p*
History of COVID-19 infection	Colombia vs. South/Central America	0.14	*
Perceived training impediment	Colombia vs. South/Central America	–0.42	*
Rehabilitation requirement	Other country vs. Colombia	–0.27	***
Rehabilitation requirement	Other country vs. South/Central America	–0.26	*
Training aided recovery	Colombia vs. South/Central America	–0.61	*

Note: * *p* < 0.05; *** *p* < 0.001. MD = mean difference; *p* = significance.

**Table 8:** Differences by municipality.

Variable	Comparison	MD	*p*
Fear of running in large crowds	Medellín vs. other municipality	–0.14	**
Training aided recovery	Medellín vs. other municipality	–0.33	***
Training aided recovery	Medellín vs. foreign country	–0.71	**
Training perceived as protective	Medellín vs. other municipality	–0.42	***
Training perceived as protective	Medellín vs. foreign country	–0.58	*

Note: * *p* < 0.05; ** *p* < 0.01; *** *p* < 0.001. MD = mean difference.

## Data Availability

The original contributions presented in this study are included in the article. Further inquiries can be directed to the corresponding author.
